# Two-Dimensional Polyacrylamide Gel Electrophoresis Coupled with Nanoliquid Chromatography–Tandem Mass Spectrometry-Based Identification of Differentially Expressed Proteins and Tumorigenic Pathways in the MCF7 Breast Cancer Cell Line Transfected for Jumping Translocation Breakpoint Protein Overexpression

**DOI:** 10.3390/ijms241914714

**Published:** 2023-09-28

**Authors:** Madhuri Jayathirtha, Taniya Jayaweera, Danielle Whitham, Brîndușa Alina Petre, Anca-Narcisa Neagu, Costel C. Darie

**Affiliations:** 1 Biochemistry & Proteomics Laboratories, Department of Chemistry and Biomolecular Science, Clarkson University, 8 Clarkson Avenue, Potsdam, NY 13699, USA; jayathm@clarkson.edu (M.J.); jayawetm@clarkson.edu (T.J.); whithad@clarkson.edu (D.W.); bpetre@clarkson.edu (B.A.P.); 2 Laboratory of Biochemistry, Department of Chemistry, “Alexandru Ioan Cuza” University of Iasi, Carol I Bvd., No. 11, 700506 Iasi, Romania; 3 Center for Fundamental Research and Experimental Development in Translation Medicine—TRANSCEND, Regional Institute of Oncology, 700483 Iasi, Romania; 4 Laboratory of Animal Histology, Faculty of Biology, “Alexandru Ioan Cuza” University of Iasi, Carol I Bvd., No. 20A, 700505 Iasi, Romania; aneagu@uaic.ro

**Keywords:** BC, JTB^high^, MCF7, DEPs, tumorigenic pathways

## Abstract

The identification of new genes/proteins involved in breast cancer (BC) occurrence is widely used to discover novel biomarkers and understand the molecular mechanisms of BC initiation and progression. The jumping translocation breakpoint (*JTB*) gene may act both as a tumor suppressor or oncogene in various types of tumors, including BC. Thus, the JTB protein could have the potential to be used as a biomarker in BC, but its neoplastic mechanisms still remain unknown or controversial. We previously analyzed the interacting partners of JTB^high^ protein extracted from transfected MCF7 BC cell line using SDS-PAGE complemented with in-solution digestion, respectively. The previous results suggested the JTB contributed to the development of a more aggressive phenotype and behavior for the MCF7 BC cell line through synergistic upregulation of epithelial–mesenchymal transition (EMT), mitotic spindle, and fatty acid metabolism-related pathways. In this work, we aim to complement the previously reported JTB proteomics-based experiments by investigating differentially expressed proteins (DEPs) and tumorigenic pathways associated with JTB overexpression using two-dimensional polyacrylamide gel electrophoresis (2D-PAGE). Statistically different gel spots were picked for protein digestion, followed by nanoliquid chromatography–tandem mass spectrometry (nLC-MS/MS) analysis. We identified six DEPs related to the JTB^high^ condition vs. control that emphasize a pro-tumorigenic (PT) role. Twenty-one proteins, which are known to be usually overexpressed in cancer cells, emphasize an anti-tumorigenic (AT) role when low expression occurs. According to our previous results, proteins that have a PT role are mainly involved in the activation of the EMT process. Interestingly, JTB overexpression has been correlated here with a plethora of significant upregulated and downregulated proteins that sustain JTB tumor suppressive functions. Our present and previous results sustain the necessity of the complementary use of different proteomics-based methods (SDS-PAGE, 2D-PAGE, and in-solution digestion) followed by tandem mass spectrometry to avoid their limitations, with each method leading to the delineation of specific clusters of DEPs that may be merged for a better understanding of molecular pathways and neoplastic mechanisms related to the JTB’s role in BC initiation and progression.

## 1. Introduction

Currently, the identification of new “key genes” and their association with breast cancer (BC) occurrence is widely used to discover new biomarkers and molecular therapeutic targets to understand the molecular mechanisms of BC progression based on cancer-associated gene/protein expression profiles that could be reliable in clinical studies [1]. Jumping translocation breakpoint (JTB), also known as the prostate androgen receptor (PAR), was described as a transmembrane protein gene at 1q21 rearranged in an jumping translocation (JT)—a rare chromosome aberration involved in various types of cancers, including BC [2,3,4]. Moreover, recurrent gains in 1q have also been reported in invasive micropapillary carcinomas (IMPC) of the breast [5], a rare type of BC characterized by lymphovascular invasion and inaccurate imaging estimation [6]. The JTB gene belongs to the epidermal differentiation complex (EDC) expressed in epithelial cells, following different patterns of cell-type specific expression [3,7]. The EDC is a cluster of 62 coding-related genes that span a 2 Mb region, encoding structural and regulatory proteins responsible for epithelial tissue development and repair [8,9]. These coding genes are present in four gene families: filaggrin (*FLG*) and FLG-like, late cornified envelope genes (*LCEs*), small proline-rich regions (*SPRRs*), and *S100* genes [10]. Many of these genes have also been associated with numerous cancers, such as skin, gastric, head and neck, colorectal, lung, ovarian, and renal carcinomas [11]. JTB gene amplification has also been associated with many malignancies, such as sarcomas, BC, and other solid tumors [12].

JTB is expressed in normal tissues from different organs and systems: the nervous system, reproductive system, digestive system, respiratory system, endocrine and exocrine glands, and urinary system, as well as in adipose tissue, leukocytes, and spleen [12,13]. Thus, JTB was found to be upregulated in many tumor tissues, such as primary BC, compared to normal counterparts, ovary, lung, uterus, colon, rectum, thyroid, prostate, stomach, kidney, and small intestine cancer [13], as well as in leukemia [3] and hepatocellular carcinoma (HCC) [14]. In addition, JTB was also upregulated in different cancer cell lines, such as MCF7 and T47D BC cell lines compared to MCF10 normal breast epithelial cell lines or in androgen-sensitive and resistant prostate cancer cell lines compared to normal prostate epithelial cell lines and normal prostatic tissue [12,13]. In these malignancies, JTB emphasizes oncogenic activity, leading to the malignant transformation of cells [13].

JTB may influence cell proliferation in vitro, clonogenicity, and in vivo tumorigenicity, being involved in the dysregulation of cell cycle progression, chromosome segregation, spindle formation, and cytokinesis [12]. Also, JTB overexpression depresses the membrane potential of mitochondria and cellular growth and conferred resistance to apoptosis induced by TGF-β1, contributing to the tumorigenic process [2]. The role of JTB in tumorigenesis is still controversial. Several malignancies from different organs suppress JTB expression, emphasizing a role in the neoplastic transformation of cells [2]. Thus, JTB expression detected in the lung, kidney, stomach, and colon decreased significantly in tumor samples compared to normal samples [2]. JTB gene expressions have been obtained using isolation and sequencing of JTB cDNA, RNA dot blot, flow cytometry (FC), Northern blot (NB), Western blot (WB), immunoprecipitation (IP), immunofluorescence microscopy (IF), transmission electron microscopy (TEM), fluorescence in situ hybridization (FISH), apoptosis assays, Aurora A kinase assays, and luciferase reporter gene assays [2,4,12]. Our previous results, based on Sodium dodecyl-sulfate polyacrylamide gel electrophoresis (SDS-PAGE) followed by nLC-MS/MS proteomics, suggested that JTB overexpression in MCF7 BC cell line may be associated with mitotic spindle assembly, late estrogen response, epithelial–mesenchymal transition (EMT), and early estrogen response pathways sustained by the deregulation of cytoskeletal organization and biogenesis, mitotic spindle organization, extracellular matrix (ECM) remodeling, cellular response to estrogen, proliferation, migration, metastasis, increased lipid biogenesis, endocrine therapy resistance, tumor microenvironment (TME) acidification, transmembrane transport, glycolytic flux, iron metabolism, oxidative stress (OS), metabolic reprogramming, nucleo-cytosolic mRNA transport, transcriptional activation, chromatin remodeling, modulation of cellular death pathways, and cancer drug resistance as biological processes [15]. We also concluded that JTB dysregulation, assessed by in-solution-based proteomics, may promote a more aggressive phenotype and behavior for MCF7 cells by synergistic upregulation of EMT, mitotic spindle organization, and fatty acid metabolism-related pathways [16].

In this work, we aim to investigate the differentially expressed proteins (DEPs) and tumorigenic pathways associated with JTB^high^ using 2D-PAGE coupled with nLC-MS/MS proteomics of MCF7 BC cell line in order to complete and complement our previously published results based on SDS-PAGE [15] and in-solution proteomics of MCF7 cells transfected for JTB upregulation [16].

## 2. Results

Of 40 DEPs identified by 2D-PAGE coupled with nLC-MS/MS proteomics, 9 were significantly upregulated, and 31 were downregulated in the MCF7 BC cell line transfected for JTB overexpression. We only analyzed the proteins that have a protein score of above 40 and *p*-value < 0.05. Thus, RBBP4, SET, ABRACL, NCKAP1, JUP, DSPI, APC, DIABLO, and HINT2 are overexpressed, while UBA1, YWHAZ, GSN, ITGB5, HSPB1, HNRNPK, PCBP2, MCCC2, UGDH, TPI1, ATP5F1B, DLST, FTL, HYOU1, PRDX6, NRDC1, PRDM5, DKK1, NCKAP1, RPL7A, RPL31, RPS3, TUBA1A, TUBA1B, TUBA1C, TUBA8, TUBA3E, TUBB, TUBB4b, TUBB3, and TUBB2A were found to be significantly downregulated. Gene Set Enrichment Analysis (GSEA) was performed for the upregulated JTB condition using H (hallmark gene sets) collection in MSigDB. Analysis of H collection revealed two upregulated pathways, including proteins important for epithelial-to-mesenchymal transition (EMT) and hypoxia. Two downregulated pathways comprised proteins involved in the mitotic spindle and unfolded protein response (UPR) pathways (Table 1). Sixteen downregulated DEPs with anti-tumorigenic potential were submitted for protein–protein interactions (PPI) network construction with Search Tool for the Retrieval of Interacting Genes/Proteins (STRING) database (https://string-db.org/, accessed on 19 September 2023) to emphasize the specific interaction network associated with JTB^high^ condition in MCF7 BC cell line. A total of 16 nodes and 26 edges were mapped in the PPI network, with an average node degree of 3.25 and an average local clustering coefficient of 0.515.

To emphasize the role of the JTB-interactome, we analyzed the pro-tumorigenic (PT) and anti-tumorigenic (AT) functions of these proteins, the neoplastic dysregulated pathways, and the biological processes (Table 2).

## 3. Discussion

### 3.1. Pro-Tumorigenic (PT) JTB-Interacting Partners

In our previous JTB^high^-related study based on SDS-PAGE followed by nLC-Ms/MS proteomics, GSEA identified several overexpressed pathways involved in mitotic spindle assembly, late estrogen response, epithelial–mesenchymal transition (EMT), and early estrogen response [15]. The main upregulated DEPs involved in this pathway were actinins (ACTNs), filamin A and B (FLNA, FLNB), ezrin (EZR), HSPA1A, HSP90A, myoferlin (MYOF), epiplakin (EPPK1), COL3A1, COL11A1, fatty acid synthase, and FOXA [15]. The study based on in-solution proteomics emphasized that JTB dysregulation is associated with the EMT process, mitochondrial organization and function, oxidative stress (OS), apoptosis, interferon alpha, and gamma signaling pathways [16].

In this experiment, of nine upregulated JTB partners, four proteins may emphasize pro-tumorigenic (PT) functions: retinoblastoma-binding protein 4/histone-binding protein (RBBP4), SET nuclear proto-oncogene/template-activating factor-I β/inhibitor of histone acetyltransferase (SET), actin-binding Rho-activating C-terminal-like (ABRACL) and noncatalytic region of tyrosine kinase (Nck)-associated protein 1 (NCKAP1) (Table 3). These upregulated proteins have been reported as overexpressed in different types of tumors, including BC tissue samples and cell lines, with all of them being involved in the EMT pathway. The EMT process was found to be upregulated in our published analysis conducted in the MCF7 BC cell line transfected for JTB overexpression, using SDS-PAGE [15], as well as in an in-solution digestion [16]. RBBP4 and SET are multitasking histone/DNA-binding proteins, which are also involved in chromatin remodeling/organization, DNA replication and repair, transcriptional regulation, histone modification, and cell cycle and promoting cell proliferation, migration, invasion, and anti-apoptosis. ABRACL is known as a regulator of the actin cytoskeleton, cell motility, and cell cycle. Acting as a cytoskeleton/actin dynamics regulator, the non-catalytic region of tyrosine kinase (Nck)-associated protein 1 (NCKAP1) is a part of the WAVE complex that regulates lamellipodia/invadopodia formation and cell mobility, sustaining cell migration. Thus, NCKAP1 promotes EMT activation [31] by HSP90-mediated invasion and metastasis, provoking MMP9 and MMP14 activation [32]. Retinoblastoma-binding protein 4 (RBBP4/RbAp48), also known as histone-binding protein RBBP4, is a 48 kDa tumor-specific/oncogenic protein involved in transcription regulatory complexes that control cell cycle gene expression [18], proliferation, migration, invasion, and apoptosis, playing an important role in chromatin metabolism, nucleosome assembly, and histone modification [17,20]. RBBP4 was identified as significantly overexpressed in human embryonal and glial brain cancers [18], BC [17], especially in TNBC tissues and cell lines [19], as well as in colon cancer cell lines, promoting malignant progression via increasing activity of the Wnt/β-catenin pathway, in correlation with a high expression of the histone deacetylase 1 (HDAC1) [20]. RBBP4 inhibition reduced cell invasion and migration through the regulation of proteins related to the EMT process in colon cancer [20], as well as in TNBC cell lines [19]. SET protein, also known as template activating factor-I β (TAF-Iβ), is involved in the cell cycle, migration, apoptosis, transcription, and DNA repair [21]. SET was found to be overexpressed in 50–60% of BC tumor samples and BC cell lines [22]. In pancreatic cancer (PDAC) progression, the various isoforms of this multifunctional onco-protein sustain EMT by inducing the transcriptional activation of the mesenchymal biomarker N-cadherin, resulting in the promotion of mesenchymal cell characteristics, proliferation, migration, invasion, and colony formation [23]. SET overexpression also activated the Rac1/JNK/c-Jun and MEK/ERK signaling pathways that lead to cell migration and proliferation [23]. The actin-binding Rho-activating C-terminal-like (ABRACL) protein, which was previously named HSCP280, is a regulator of the actin cytoskeleton, cell motility, and viability [24] through its interaction with cofilin [28]. It is highly expressed in several cancers, such as endometrial cancer [26], BC tissues and cells [25], colon cancer cells [28], esophageal carcinoma [27], and gastric cancer [24]. ABRACL knockdown suppressed the proliferation, invasion, migration, and EMT of BC cells [25].

Of 23 downregulated JTB partners, two proteins may emphasize PT functions: gelsolin (GSN) and PRDI-BF1 and RIY domain containing 1 (PRDM5/PFM2) (Table 3). GSN is an actin-binding protein downregulated in CC, HCC, GC, cervical cancer, ovarian cancer [64], and BC [65,66]. It is known that actin-remodeling proteins have an important role in the regulation of cytoskeletal as well as inflammatory cell responses [129]. GSN knockdown leads to EMT in mammary epithelial cells [67]. GSN deficiency increases with progression from atypical ductal hyperplasia (ADH) to ductal carcinoma in situ (DCIS) to invasive breast cancer (IBC) [66]. Mukherjee et al. (2012) have shown that GSN knockdown in 3T3-L1 adipocyte cells suppressed the expression of lipogenic genes but highly increased that of tumor necrosis factor-α (TNFα) and inflammatory interleukin-6 (IL-6) [130]. IL-6 is known to be significantly produced by BC cellsand cancer-associated adipocytes (CAAs), with the potential of inducing proliferation, EMT, stem cell phenotype, angiogenesis, cachexia, and therapeutic resistance in BC cells [131], correlating with clinical disease stage and lymph node metastasis as well as with ER and HER2 expression [132]. Moreover, IL-6 is an important growth factor for estrogen receptor-α (ERα)-positive BC [133]. Additionally, TNFα is a pro-inflammatory cytokine that was reported as overexpressed in BC, where it correlates with augmented cancer cell proliferation, increased metastasis, higher malignancy grade, and poor prognosis [134]. The exposure of the MCF7 BC cell line to a low dose of TNFα enhanced the invasive phenotype by influencing the different genes involved in metastasis [135]. PRDM5/PFM2 is a zinc finger protein acting as an epigenetic modifier [113] known as a tumor suppressor; it is frequently silenced/downregulated/inactivated by methylation in multiple carcinoma lines, such as NPC, ESCC [114], GC [115], and HCC and ovarian, cervical, and breast cancers [113,116]. PRDM4 knockdown increases cell growth, proliferation, migration, invasion, tumor initiation, and progression [113,114,115], emphasizing an opposite role in melanoma [116].

### 3.2. Anti-Tumorigenic (AT) JTB-Interacting Partners

Two upregulated JTB-related partners, histidine triad nucleotide-binding protein (HINT2) and Armadillo repeats domain of adenomatous polyposis coli (APC), have been detected in this experiment (Table 4). The upregulated APC might emphasize AT functions. It is known as a multi-functional tumor suppressor protein that regulates cell–cell adhesion, cell polarization, migration [43], cell proliferation and differentiation [44], organization of actin and microtubule networks, spindle formation, and chromosome segregation [45]. APC was reported as mutated in colon cancer (CC) and liver cancer, where it acts as a negative regulator of canonical Wnt signaling [45]. HINT2 is a tumor suppressor protein present in the mitochondrial matrix that sensitizes cells to apoptosis [50] and positively regulates lipid and glucose metabolism as well as mitochondrial respiration [51]. HINT2 overexpression induces an anti-EMT gene expression profile in cancer cells, inhibiting cell migration, invasion, and metastasis [52]; induces cell apoptosis; decreases mitochondrial membrane potential; promotes intracellular ROS production; and elevates mitochondrial Ca^2+^ levels [53].

**Figure 1:**
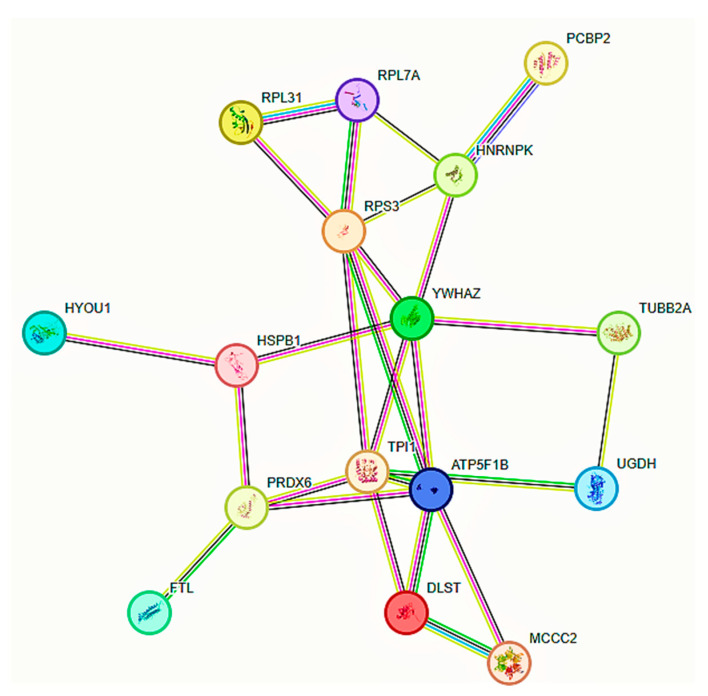
Protein–protein interactions (PPI) network of anti-tumorigenic (AT) DEPs in MCF7 BC cell line transfected for JTB upregulation, by means of STRING on-line database (https://string-db.org/, accessed on 19 September 2023). A total of 16 nodes and 26 edges were mapped in the PPI network. RPL31—60Sribosomal protein L31; RPL7A—60S ribosomal protein L7a; RPS3—40S ribosomal protein S3; PCBP2—poly(rC)-binding protein 2; HNRNPK—heterogeneous nuclear ribonucleoprotein K; HYOU1—hypoxia upregulated 1; HSPB1—heat shock protein beta-1; YWHAZ—tyrosine 3-monooxygenase; TUBB2A—tubulin beta-2A chain; FTL—ferritin light chain; PRDX6—peroxiredoxin 6; TPI1—triosephosphate isomerase 1; ATP5F1B—adenosin triphosphate synthase F1 subunit beta; UGDH—UDP-glucose 6-dehydrogenase; MCCC2—methylcrotonyl-CoA.

Several downregulated JTB^high^-related proteins exert AT functions (Table 4). Sixteen downregulated DEPs with anti-tumorigenic potential (YWHAZ, TUBB2A, HSPB1/HSP27, HNRNPK, PCBP2, MCCC2, UGDH, TPI1, ATP5F1B, DLST, FTL, HYOU1, PRDX6, RPL31, RPL7A, and RPS3) were submitted for protein–protein interaction (PPI) network construction with the STRING database (https://string-db.org/, accessed on 19 September 2023) to emphasize the specific interaction network associated with JTB^high^ condition in the MCF7 BC cell line (Figure 1). HSP27/HSPB1 is known to be overexpressed in BC [72]. HSP27-downregulated cells, however, showed a significant increase in the expression of phosphatase and tensin homolog (PTEN), which is a tumor suppressor gene that is deleted in many tumors [72]. Using reverse-phase (RP)-nLC-ESI-MS/MS analysis, Shin et al. showed that tubulin beta-2A chain (TUBB2A) may be considered as a biomarker for the prediction of distant metastatic BC, based on this high expression in highly invasive BC cell lines [63]. Thus, by siRNA transfection, TUBB2A was downregulated, inducing a decreased invasiveness/migration ability of BC cells [63]. Ubiquitin-like modifier-activating enzyme 1 (UBA1) silencing inhibits cell proliferation, migration, and invasion; upregulates proteolytic and DNA damage stress, the Fe^2+^ content in cells, and ferroptosis; suppresses the NRF2 signaling pathway [57]; elicits UPR; induces cell death [60]; has high toxicity for TNBC models; upregulates ER stress; and upregulates the pro-apoptotic phorbol-12-myristate-13-acetate-induced protein (PMAIP1), also known as NOXA [58]. Tyrosine 3-monooxygenase/tryptophan 5-monooxygenase activation protein zeta (YWHAZ/14-3-3ζ) is an oncogene overexpressed in multiple cancers, such as HCC, CRC, LUAD, BC [61], and urothelial carcinomas [62]. Its knockdown decreases cell growth, proliferation, and invasion and enhances apoptosis and tamoxifen-induced inhibition of cell viability [61]. Integrin subunit beta 5 (ITGB5) is an integral transmembrane protein involved in cell adhesion, known as an oncogenic factor overexpressed in aggressive tumors, such as primary and metastatic TNBC [68], GBM [69], and CRC [70]. ITGB5 depletion reduced tumor growth, survival, proliferation, migration, invasion, and angiogenesis [68,69,70]. Poly(rC)-binding protein 2 isoform b (PCBP2) is an RNA-binding protein that contributes to transcriptional and translational regulation [81,82]. It is cited as an oncogene that promotes GC [81], HCC, and GBM, being also detected in BC tissues and cell lines [83]. PCBP2 depletion decreases GC cell viability and proliferation [81] and inhibits cell proliferation, colony formation, migration, invasion, in vivo tumor growth, and metastasis in BC [83]. As a mitochondrial member of the biotin-dependent carboxylase superfamily, the methylcrotonyl-CoA carboxylase 2 (MCCC2) is an oncogene overexpressed in HCC [84], BC [85], PCa [86], and CRC [87]. MCCC2 knockdown reduces cell proliferation, migration, and invasion; glycolysis markers; glucose consumption; lactate secretion; acetyl-CoA levels [84]; and promotes apoptosis [85]. UDP-glucose 6-dehydrogenase (UGDH) is a metabolic enzyme associated with mesenchymal-like gene expression [88]. UGDH has been found to be upregulated in epithelial cancers, including BC [88], highly metastatic ovarian cancer cell lines [89], GBM [90], and lung cancer [91]. UGDH knockdown decreases cell motility, invasion, glycosaminoglycan biosynthesis, cell migration [90], tumor growth, hyaluronic acid production, and colony formation [88] and induces cell cycle arrest in the G_0_/G_1_ phase [89]. Another glycolytic enzyme involved in metabolic reprogramming, triosephosphate isomerase 1 (TPI1), exerts oncogenic functions when translocated to the cell nucleus induced by stress condition. TPI1 is overexpressed in multiple cancers, such as BC tissues and cell lines [92] or LUAD [93]. According to the GSEA database, this enzyme is involved in MTORC1_SIGNALING, GLYCOLYSIS, and HYPOXIA, as well as in EMT pathways [92]. Its knockdown reduces cell migration, colony formation, and xenograft tumor growth [93]. Another metabolic enzyme, adenosine triphosphate synthase F1 subunit beta, mitochondrial precursor (ATP5F1B/ATP5B), is present in the inner mitochondrial membrane. It is involved in ATP synthesis via OXPHOS and is ectopically expressed on the surface of various cancer cells [94]. This enzyme was cited as a participant in carcinogenesis in several tumors, being overexpressed in BC, especially in luminal and HER2+ subtypes [94] and the plasma membrane of highly invasive cells, including MDA-MB-231 BC cells [95] and GC [96]. When overexpressed, this enzyme increases intracellular ATP in cancer cells, promoting migration, invasion [95], and proliferation [94], while its inhibition suppresses cancer cell metastasis and growth [96]. Dihydrolipoamide S-succinyltransferase (DLST) is an oncoprotein highly expressed in BC, including MCF7, MDA-MB-231 BC cell lines [97], and TNBC [99] as well as in neuroblastoma [98]. DLST depletion impedes cancer initiation and progression, impairs OXPHOS, suppresses cell growth and TCA-cycle, increases ROS levels, induces apoptosis, and decreases cancer invasiveness [98,99]. Another oncogene overexpressed in various malignant tumors [101], such as GBM cells and serum [100] and CRC tissues and cell lines [102], ferritin light chain (FTL), interacts with PI3K/Akt, GADD45/JNK, TGF-β signaling, and cell cycle proteins. FTL knockdown decreases the expression of Wnt target genes, cyclin D1, and c-Myc [100] and represses EMT by regulation of Akt/GSK_3_β/β-catenin signaling [101]. Additionally, FTL silencing inhibits cancer cell growth, viability, and proliferation [100,103]; reduces cancer cell survival, migration, and invasion; and increases apoptosis [101]. Hypoxia upregulated 1 (HYOU1/GRP170/ORP150) is a molecular chaperone that belongs to the HSP70 protein family, with a cytoprotective role involved in protein folding in ER under stressful conditions [104,105]. It was reported as upregulated in many cancers, such as BC, papillary thyroid carcinoma (PTC), nasopharyngeal cancer (NPC), epithelial ovarian cancer (EOC), and Kaposi’s sarcoma (KS) [105]. Gene silencing promotes OXPHOS and inhibits aerobic glycolysis [105], suppressing cancer cell proliferation, migration, and invasion [105]. Peroxiredoxin 6 (PRDX6), an antioxidant enzyme involved in the ROS pathway, is overexpressed in various cancers [106], such as cervical cancer [107], CRC [108], as well as in MDA-MB-231 HM BC cell line [109]. Its knockdown inhibits cancer cell proliferation, migration, and invasion and stimulates apoptosis [107]. Nardilysin convertase 1 (NRDC1) is a nuclear, cytoplasmic, or cell-surface zinc-dependent endopeptidase of the M16 family that acts as a transcriptional co-regulator [110], cell-surface receptor for heparin-binding EGF-like factor (HB-EGF), emphasizing an epigenetic regulatory function [111]. Its deficiency diminishes tumor size, suppresses carcinogenesis/proliferation [111], spheroid growth, and signal transducer and activator of transcription 3 (STAT3) phosphorylation [110]. The Dickkopf Wnt signaling pathway inhibitor 1 (DKK1) is a secretory antagonist of the β-catenin-dependent Wnt signaling pathway [117]. DKK1 has been reported as a tumor suppressor in colon cancer (CC) [118] or as an oncogene abnormally expressed in tumor cells or overexpressed in many cell lines and HCC [119], lung cancer, BC, cervical cancers, and glioma [117]. Consequently, the gene knockdown may inhibit migration, invasion, proliferation, cancer stem-cell-like proprieties, tumor growth, and angiogenesis and enhance apoptosis and tumor regression [117]. NCK-associated protein 1, isoform CRA_a (NCKAP1/NAP1), is a member of the Wiskott–Aldrich syndrome protein family member (WASF) regulatory complex (WRC) involved in actin cytoskeleton organization, lamellipodia formation, cell motility, and adhesion [32]. It was reported as overexpressed in high-grade tumors, including BC, PCa, CC [120], and NSCLC tissue [32]. NCKAP1 silencing destabilizes the WASF3 complex involved in actin cytoskeletal reorganization, cell movement, and invasion [121], suppressing invasiveness and metastasis [120] and reducing MMP9 secretion [32]. The heterogeneous nuclear ribonucleoprotein K (HNRNPK) is a multifunctional RNA-binding protein (RBP) that contributes to chromatin remodeling, transcription, splicing, and translation [75].

Ribosomal proteins (RPs) are involved in tumorigenesis and cell transformation. The knockdown of many RPs, such as RPS3, RPS15A, RPL39, and RPS6, is shown to be linked to a reduction in BC cell growth, proliferation, viability, or metastasis [126]. Overexpression is present in BC, including TNBC, especially metastatic TNBC cells, as well as in PCa cell lines [122,124]. 60S ribosomal protein L7a (RPL7A) blocking may reduce cell migration and invasion [123]. Additionally, identified as downregulated in the present JTB-overexpressed condition, 60S ribosomal protein L31 (RPL31) and 40S ribosomal protein S3 (RPS3) may enhance the levels of p53 and p21, decreasing cell growth and cell cycle [125], by promoting ribosomal stress, which impairs ribosomal biogenesis; impedes cell proliferation, invasion, and migration; and increases apoptosis [127].

### 3.3. JTB-Interacting Partners with Controversial Neoplastic Functions

The second mitochondrial-derived activator of caspase (SMAC)/direct IAP-binding protein with low pI (DIABLO) was found to be overexpressed in this experiment. DIABLO is known as a promoter of caspase-dependent apoptosis (HALLMARK_APOPTOSIS) by inhibition of inhibitory apoptotic proteins (IAP) family members. As a promoter of apoptosis, DIABLO is known as an AT protein released from mitochondria into the cytosol in response to the apoptotic stimuli [136]. DIABLO has a dysregulated expression in many cancers [137]. There are studies that reported DIABLO as overexpressed in GC, CRC, and ovarian cancer and as downregulated in PCa, lung, and soft tissue cancers, as well as in BC tissues, with its expression decreasing with BC progression [47]. However, DIABLO was recently found to be involved in non-apoptotic processes that are essential for tumor growth and progression [49], such as phospholipid synthesis associated with tumorigenesis [48]. Thus, other published works reported DIABLO overexpression in BC, lung, bladder, cervical, pancreas, prostate, melanoma, glioma [48,49], ER-positive BC cell lines, such as MCF7 that is an ER-positive and PR-positive luminal A BC subtype [138], as well as in ER-positive BC in comparison with ER-negative tissue samples, demonstrating a poor prognosis in BC patients [137]. There is evidence that the overexpression of DIABLO increased the mammosphere-forming ability of MCF7 BC cells and activation of the cell survival and proliferation pathways, while it is known that apoptosis itself may induce proliferation and invasion of more aggressive cancer cells to induce tumor growth and expansion [137]. The upregulation of DIABLO in the MCF7 BC cell line in the JTB overexpressed condition may be correlated with an increase in aggressiveness and invasion abilities of this BC cell line comparable with the luminal B BC cell lines that show an increased expression of proliferation-related genes and a higher risk of early relapse compared with the luminal A subtype [137,139]. Recently, based on proteomics–interactomics-based analysis and a lung cancer mouse model, DIABLO depletion was correlated with the inhibition of tumor cell growth and proliferation, decreased phospholipid levels, activated apoptosis, reversed EMT and altered TME, reduced expression of inflammation-related proteins such as NF-κB and TNF-α, and of the programmed death-ligand 1 (PD-L1), which is associated with immune system suppression [49]. The expression of DIABLO inversely correlates with the BC tumor stage, suggesting that this protein may play an important role in BC development [47].

Junction plakoglobin (JUP) and desmoplakin (DSP) are also upregulated in this experiment. DSP is a cell–cell adhesion molecule reported to have an AT activity in non-small-cell lung cancer (NSLC) by inhibition of the Wnt/β-catenin signaling pathway, followed by inhibition of cell proliferation, migration, and invasion and an increase in sensitivity to induced apoptosis [40]. Loss of DSP has been involved in BC metastasis [140]. JUP, also known as γ-catenin, a member of the Armadillo family of proteins and a paralog of β-catenin, is a component of both desmosomes, in association with DSP, and adherent junctions, along with β-catenin and α-catenin, playing an essential role in the regulation of cell–cell adhesion and participating in cell signaling [141]. JUP overexpression was reported in many cancers [34], where it may act as an oncogenic or tumor suppressor protein [36]. There are studies focused on various mechanisms by which plakoglobin may inhibit tumorigenesis and metastasis [141]. However, other works showed that the aberrant activation of γ-catenin promotes genomic instability and oncogenic effects during tumor progression [34]. Also, the overexpression of plakoglobin was able to promote cell survival and metastasis in invasive micropapillary carcinoma of the breast (IMPC) by activation of the PI3K/Akt/Bcl-2 pathway, which may protect the clusters of CTCs from anoikis [35]. JUP, as well as DSP, are barrier molecule genes (BMGs) that encode proteins that mediate the mechanical barrier function in normal epithelia, whose overexpression was associated with decreased immune surveillance and shorter patient survival [142]. Both JUP and DSP were identified to be upregulated in melanoma cell lines, where they significantly increase tumor burden, VEGF-A, reduced IL-33, and angiogenesis [41], thus emphasizing their PT roles.

## 4. Materials and Methods

The MCF7 cell culture, the transfection of hJTB plasmids, and the collection of cell lysates were described previously [15] and briefly described below.

### 4.1. Cell Culture

MCF7 cells were purchased from American Type Culture Collection (HTB–22 ATCC) and grown in RPMI-1640 medium supplemented with 10% FBS, 1% Penicillin Streptomycin, 0.2% Gentamycin, and 0.2% of Amphotericin (growth media) at 37 °C and in 5% CO_2_. The cells were grown until they reached ~70% confluency and transfected with JTB cDNA plasmid for overexpression.

### 4.2. Plasmids for Upregulation

Plasmids were custom-made by Genscript^®^_._ One plasmid with the hJTB gene containing the full coding region of cDNA, ggtaccGCCACCATGCATCATCATCATCATCATCTTGCGGGTGCCGGGAGGCCTGGCCTCCCCCAGGGCCGCCACCTCTGCTGGTTGCTCTGTGCTTTCACCTTAAAGCTCTGCCAAGCAGAGGCTCCCGTGCAGGAAGAGAAGCTGTCAGCAAGCACCTCAAATTTGCCATGCTGGCTGGTGGAAGAGTTTGTGGTAGCAGAAGAGTGCTCTCCATGCTCTAATTTCCGGGCTAAAACTACCCCTGAGTGTGGTCCCACAGGATATGTAGAGAAAATCACATGCAGCTCATCTAAGAGAAATGAGTTCAAAAGCTGCCGCTCAGCTTTGATGGAACAACGCTTATTTTGGAAGTTCGAAGGGGCTGTCGTGTGTGTGGCCCTGATCTTCGCTTGTCTTGTCATCATTCGTCAGCGACAATTGGACAGAAAGGCTCTGGAAAAGGTCCGGAAGCAAATCGAGTCCATAGACTACAAAGACGATGACGACAAGTACCCATACGATGTTCCAGATTACGCTgatatc, corresponding to 146 amino acids of the protein, was used. The hJTB cDNA in sense orientation was inserted into a CMV promoter-based plasmid for JTB overexpression. The plasmid was further customized to have three tags His, HA, and FLAG tags. It also had an eGFP tag to enable confirmation of transfection in MCF7 cells. The second plasmid was an empty vector with an eGFP tag to serve as a control.

### 4.3. Transfection into MCF7 Cells

Lipofectamine™ 3000/DNA and DNA/Plasmid (10 µg/µL) complexes were prepared in Opti-MEM Reduced Serum Media (Invitrogen, Eugene, OR, USA) for each condition and added directly to the cells in culture medium. A total of 2 mg/mL of Neomycin was added after 48 h and incubated at 37 °C. Cells that survived were allowed to reach 80% confluency by replacing the growth media every 48 h with new media containing 2 mg/mL antibiotic. Transfection efficiency was confirmed by visualizing the green fluorescence emitted by the eGFP gene using a confocal microscope.

### 4.4. Western Blot Analysis

Cell lysates from each condition were collected using a lysis buffer containing 20 mM Tris HCl, 150 mM Nacl, 0.2 mM EDTA, 1.1% Triton-X, and protease/phosphatase inhibitors. The lysates were then incubated for 30 min on ice and centrifuged at 14,000 rpm for 20 min at 4 °C. The supernatants were collected, and protein concentration was determined using Bradford assay with bovine serum albumin standards. Lysates containing 20 µg of proteins were run in 14% SDS-polyacrylamide gels and transferred to nitrocellulose membranes. The blots were incubated with blocking buffer containing 5% milk and 0.1% tween-20 overnight at 4 °C with shaking. Primary antibody (JTB Polyclonal Antibody—PA5-52307, Invitrogen) diluted to 1:1000 was added and incubated at 4 °C for 1 h with constant shaking. Secondary antibody (mouse anti-rabbit IgG-HRP sc-2357, Santa Cruz Biotechnology, Inc., Santa Cruz, CA, USA) diluted to 1:2000 ratio was added and incubated for 1 h at room temperature with constant shaking. After each incubation, the blots were washed thrice with TBS-T (1X TBS buffer, containing 0.05% tween-20) for 10 min each with constant shaking. Finally, the enhanced chemiluminescence (CL) substrate (Pierce™ ECL Western Blotting Substrate—32106, Thermo Fisher Scientific, Waltham, MA, USA) was added to the blot, and the blot was analyzed using a CCD imager. For normalization, the blots were treated with Mouse GAPDH monoclonal antibody (51332, cell-signaling technology) and incubated for 1 h, followed by 1 h incubation of goat anti-mouse IgG-HRP (sc-2005, Santa Cruz Biotechnology) and the addition of ECL substrate. Detection and comparison of the intensity of the bands were carried out using ImageJ software (National Institute of Health, Bethesda, MD, USA).

### 4.5. 2D-PAGE and Proteomic Analysis

**Figure 2:**
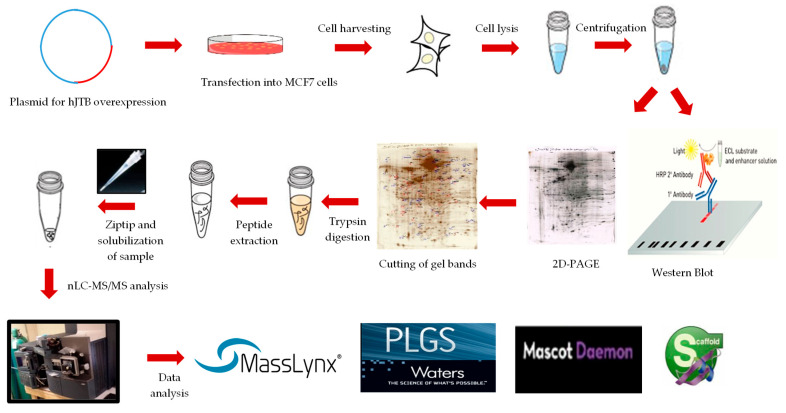
Workflow for MCF7 proteomics for 2D-PAGE.

The entire proteomics-based workflow is shown in Figure 2. We used three biological replicates of control (*n* = 3) and up_JTB (*n* = 3). These conditions were analyzed in 2D-PAGE by Kendrick Labs, Inc. (Madison, WI) and nLC-MS/MS, as previously described [15,143]. The computer comparisons were carried out for the average of control vs. Up_JTB (*n* = 3). The dysregulated spots were selected based on the criteria of having a fold increase or decrease of ≥1.7 and *p* value of ≤0.05. There were 68 dysregulated spots in control vs. up_JTB comparison (Figures S1 and S2 from Supplementary Materials) that were selected for nLC- MS/MS analysis, as previously described [143]. The data processing was carried out using ProteinLynx Global Server (PLGS) software, version 2.4, Waters Corp, Millford, MA, USA to convert them to pkl files, and Mascot Daemon software (version 2.4, Matrix Science, Boston, MA, USA) was used to identify the dysregulated proteins. The quantitative analysis of dysregulated spots was conducted using Scaffold software version 4.0 (Proteome Software, Inc, Portland, OR, USA). Gene Set Enrichment Analysis (GSEA) (https://www.gsea-msigdb.org/gsea/index.jsp, accessed on 1 December 2022) was performed to identify the dysregulated pathways as previously described [15]. Search Tool for the Retrieval of Interacting Genes/Proteins (STRING) database (https://string-db.org/, accessed on 19 September 2023) was used to construct a protein–protein interactions (PPI) network for downregulated DEPs with anti-tumorigenic functions associated with JTB^high^ condition in MCF7 BC cell line.

### 4.6. Data Sharing

Raw data from MassLynx, HTML files from Mascot, and Scaffold files will be provided upon request, according to Clarkson University Material Transfer Agreement.

### 4.7. Statistical Analysis

Data are presented as mean ± S.E.M. Statistical comparisons of three means were made using paired Student’s t-test where appropriate *p* values < 0.05 were considered statistically significant.

## 5. Conclusions

Identification of new genes/proteins involved in breast cancer (BC) occurrence is widely used to discover novel biomarkers and explain the molecular mechanisms of BC initiation and progression based on cancer-associated gene/protein expression profiles that could be reliable in clinical studies. Jumping translocation breakpoint (JTB) gene may act both as a tumor suppressor or oncogene in various types of tumors. JTB protein has been reported as overexpressed in some BC cell lines and primary breast tumors compared with their normal tissue samples counterparts, as well as in prostate and liver cancer [3,13]. Thus, JTB protein could have the potential to be used as a biomarker in BC, but its neoplastic mechanisms still remain unknown or controversial. We previously analyzed the interacting partners of JTB^high^ protein extracted from transfected MCF7 BC cell line, using SDS-PAGE [15], later complemented by in-solution digestion-based analysis [16]. The previous results suggested JTB contribution to the development of a more aggressive phenotype and behavior of MCF7 BC cell line through synergistic upregulation of epithelial–mesenchymal transition (EMT), mitotic spindle, and fatty acid metabolism-related pathways [16]. In this work, we complemented the previously reported JTB proteomics-based experiments by investigating differentially expressed proteins (DEPs) and tumorigenic pathways associated with JTB overexpression using two-dimensional polyacrylamide gel electrophoresis (2D-PAGE). Statistically different gel spots were picked for protein digestion, followed by nanoliquid chromatography–tandem mass spectrometry (nLC-MS/MS) analysis. We identified 6 differentially expressed proteins (4 upregulated and 2 downregulated) related to JTB^high^ condition vs. control that emphasize a pro-tumorigenic (PT) role (RBBP4, SET, ABRACL, NCKAP1, GSN, and PRDM5), while 21 proteins, which are known to be usually overexpressed in cancer cells, emphasized an anti-tumorigenic role when low expression occurs as happened in this experiment (UBA1, YWHAZ, TUBB2A, ITGB5, HSPB1, PCBP2, MCCC2, UGDH, TPI1, ATP5F1B, DLSI, FTL, HYOU1, PRDX6, NRDC1, and HNRNPK), as well as three ribosomal proteins (RPL7A, RPL31, and RPS3). Two upregulated proteins, APC and HINT2, also emphasize a tumor-suppressive role. According to our previous results, proteins that have a PT role are mainly involved in the activation of the EMT process. GSEA was performed for the upregulated JTB condition using H (hallmark gene sets) collection in MSigDB. Analysis of H collection revealed two upregulated pathways, including proteins important for EMT and hypoxia. Two downregulated pathways comprised proteins involved in the mitotic spindle and unfolded protein response (UPR) pathways. Interestingly, JTB overexpression has been correlated here with a plethora of significant upregulated and downregulated proteins that sustain JTB tumor suppressive functions, such as decreased cell growth, survival, motility, invasiveness and migration ability, cell proliferation, angiogenesis, colony formation, metabolism, and EMT in addition to an enhancement of apoptosis and other mechanisms of cancer cell death. These results mainly suggest the AT potential of the JTB gene. Moreover, our present and previous results sustain the necessity of complementary use of different proteomics-based methods (SDS-PAGE, 2D-PAGE, and in-solution digestion) followed by MS analysis to avoid their inherent limitations, with each method leading to the delineation of specific clusters of DEPs that may be merged together for a better understanding of molecular pathways and neoplastic mechanisms related to JTB gene or gene product role in BC initiation and progression.

## Figures and Tables

**Table 1:** Significant up- and downregulated pathways in upregulated JTB condition in MCF7 BC cell line, according to GSEA with FDR < 25%.

	Pathways	NES	FDR q-Val
Upregulated	Hypoxia	1.49	0.535
	EMT	1.08	1
Downregulated	Mitotic_spindle	−1.17	1
	UPR	−1.09	1

Abbreviations: EMT—epithelial–mesenchymal transition; FDR—false discovery rate; NES—normalized enrichment score; UPR—unfolded protein response.

**Table 2:** DEPs, neoplastic roles, and biological processes expressed in response to JTB upregulation in MCF7 BC cell line.

Gene Name	Gene Description	Role in Biological Processes	Expression in Malignancies and Putative Neoplastic Effects		Pathways	Neoplastic Behavior
Upregulated proteins						
RBBP4/RbAp48	retinoblastoma-binding protein 4/histone-binding protein RBBP4	tumor-specific, histone/DNA-binding protein involved in chromatin remodeling, DNA replication, nucleosome assembly, histone modification, transcription regulation, cell cycle and proliferation, cell differentiation and signaling, cell motility, protein-containing complex assembly [17,18]	overexpressed in embryonal and glial brain cancers [18], TNBC tissues and cell lines [19], CC cell lines [20]	PT	EMT [19], nuclear accumulation of β-catenin, activating Wnt/β-catenin signaling [20]	cell proliferation, invasion, migration, anti-apoptosis [17]
SET/TAF-I β isoform CRA_c/INHAT	SET nuclear proto-oncogene/template-activating factor-I β/inhibitor of histone acetyltransferase	multitasking histone/DNA-binding protein involved in DNA replication, chromatin organization, transcription regulation, nucleosome assembly, histone chaperoning (uniprot.org), protein-containing complex assembly, cell cycle, apoptosis, DNA repair [21]	overexpressed in 50–60% of BC [22], pancreatic cancer, leukemias, lymphomas, nephroblastoma, hepatoma, choriocarcinoma [23]	PT	EMT, Rac1/JNK/c-Jun, MEK/ERK [23]	N-cadherin activation, cell growth, proliferation, migration, invasion, colony formation [23]
ABRACL/HSCP280/ABRA C-terminal-like	actin-binding Rho-activating C-terminal-like	actin cytoskeleton remodeling, cell motility [24], cell cycle [25]	overexpressed in BC cells [25], endometrial cancer [26], esophageal carcinoma [27], GC [24], colon cancer [28]	PT	EMT [25], proteasome degradation, mitochondrial pathway [24]	proliferation, invasion, migration [25,28]
NCKAP1/NAP1	noncatalytic region of tyrosine kinase (Nck)-associated protein 1	cytoskeleton/actin dynamics regulator, part of WAVE complex that regulates lamellipodia/invadopodia formation and cell mobility [29], cell adhesion and migration [30]	overexpressed in CRC [31], NSCLC [32], BC [30]	PT	EMT activation [31] by HSP90-mediated invasion and metastasis by provoking MMP9 and MMP14 activation [32]	promotes primary tumor growth and progression to metastatic disease by directing the polarized interaction of BC cells with collagen fibrils; increases cell migration, invasion [31], and MMP9 secretion [32]
JUP/γ-catenin	junction plakoglobin	member of Armadillo family, homolog of β-catenin, common junctional plaque protein involved in cell–cell junction and signaling [33]	overexpressed in many cancers [34]: IMPC tissue and cells [35], PCa [36], AML [37], ESCC [38]	tumor suppressor (AT) in ESCC [38]; when overexpressed and located in nucleus, PT/ oncoprotein [35]	AJ; activation of PI3K/Akt/Bcl-2 and cluster cells survival [35], JUP/EGFR/AKT/GSK3β involved in tumor metastasis via inducing nuclear β-catenin translocation to upregulation of MMP7 expression in GC, promoting EMT and increasing invasion potential [39]	downregulation of CDH1, overexpression of VIM, increases cell mobility and migration, enhances pAKT and pERK, increases in PTTG and c-Myc protein levels, chromosomal instability and uncontrolled proliferation [34], tumor cluster regulator, metastasis promoter, and apoptosis downregulation [35]
DSPI//DP isoform I	desmoplakin I	tumor-suppressor; component of desmosomal plaques that interacts with JUPl; DSP overexpression enhances JUP expression [40]	inactivated in NSCLC [40], overexpressed in melanoma cell line [41]	AT or controversial, depending on the localization; in nucleus, it is involved in telomere maintenance [42]	when overexpressed, reduced expression of Wnt/β-catenin [40]	depletion induces DNA damage response and cell apoptosis, blocking the normal function of centrosomes; overexpression may have opposite effects
APC/3NMW	chain A, Armadillo repeats domain of adenomatous polyposis coli (APC)	multi-functional tumor suppressor; regulates cell–cell adhesion, cell polarization, and migration [43]; cell proliferation and differentiation [44]; organization of actin and microtubule networks, spindle formation, and chromosome segregation [45]	mutated in colon cancer and liver cancer [45]	AT	negative regulator of canonical Wnt signaling [43,45]	deregulation involved in carcinogenesis
DIABLO/ SMAC	direct IAP-binding protein with low pI/second mitochondrial-derived activator of caspase	mitochondrial protein that promotes caspase-dependent apoptosis by inhibition of IAP family members [46]	overexpressed in GC, CRC, and ovarian cancer; downregulated in PCa, lung, and soft tissue cancers; more downregulated in BC tissues than in control samples; expression decreases with BC progression [47]	AT or PT	APOPTOSIS, phospholipid biosynthetic pathways [48]	pro- and non-apoptotic functions; SMAC DEPs are associated with lipids, lipid-signaling molecules, metabolism, DNA/RNA-associated proteins, transport and intracellular trafficking, cellular signaling, immunity, TME reorganization [49]
HINT2	histidine triad nucleotide-binding protein 2, mitochondrial precursor	tumor suppressor in mitochondrial matrix; sensitizes cells to apoptosis [50]; positively regulates lipid and glucose metabolism and mitochondrial respiration [51]	downregulated in HCC [50], CRC [52], pancreatic cancer [53], BC [54]	AT	overexpression induces an anti-EMT gene expression profile in cancer cells [52]	overexpression inhibits cell migration, metastasis, and invasion [52]; induces cell apoptosis; decreases mitochondrial membrane potential; promotes intracellular ROS production; elevates mitochondrial Ca^2+^ levels [53]
**Downregulated proteins**						
UBA1	ubiquitin-like modifier-activating enzyme 1	initiation of ubiquitination cascade [55], regulator of proteostasis [56]	involved in development of HCC [57], TNBC [58], SCLC [59]	AT	ubiquitin-conjugation pathway (UCP) [59]; ferroptosis regulator [57]	silencing inhibits cell proliferation, migration, and invasion; upregulates proteolytic and DNA damage stress, the Fe^2+^ content in cells and ferroptosis; and suppresses NRF2 signaling pathway [57]; elicits UPR and induces cell death [60]; highly toxic for TNBC models; upregulation of ER stress; pro-apoptotic [58]
YWHAZ/14-3-3ζ	tyrosine 3-monooxygenase/tryptophan 5-monooxygenase activation protein zeta	central hub protein involved in many signal transduction pathways [61]	oncogene overexpressed in multiple cancers: HCC, CRC, LUAD, BC [61], urothelial carcinomas [62]	AT	UPR	knockdown decreases cell growth, proliferation, and invasion; enhances apoptosis and tamoxifen-induced inhibition of cell viability [61]
TUBB2A	tubulin beta-2A chain	associated with cellular proliferation, movement, and adhesion; involved in mitotic cell cycle, cytoskeleton organization, and cell migration; novel biomarker for the prediction of distant metastatic BC [63]	overexpressed in invasive BC cell lines [63]	AT	UPR TNFA_SIGNALING_VIA_NFKB	decreased invasiveness and cell migration [63]
GSN	gelsolin	actin-binding protein/actin regulator	dysregulated in various cancers; downregulated in CC tissues, HCC, GC, cervical cancer, ovarian cancer [64], BC [65,66]	PT	knockdown leads to EMT in mammary epithelial cells [67]	cell motility [66]; controls CDH1 to N-cadherin conversion via Snail [67]; deficiency increases with progression from ADH to DCIS to IBC [66]
ITGB5	integrin subunit beta 5	integral transmembrane protein involved in cell adhesion	oncogenic factor overexpressed in aggressive tumors: primary and metastatic TNBC cells [68]; GBM [69], CRC [70]	AT	overexpressed, mediates TGF-β/SMAD signaling and facilitates EMT in cancer cells [69,70]; deficiency leads to inhibition of Src-FAK and MEK-ERK signaling [68]	depletion reduces tumor growth, survival, proliferation, migration, invasion, and angiogenesis [68,69,70]
HSPB1/ HSP27	heat shock protein beta-1	stress-inducible chaperone	overexpressed in many cancers [71], BC tissues [72], and MCF7 cells [73]	AT	APOPTOSIS; downregulation in MCF7 cells induces upregulation of PTEN (tumor suppressor) that negatively regulates PI3K/AKT signaling [72]	negative regulator of ferroptotic cancer cell death [74] and apoptotic death [71]; HSP27 downregulation was correlated with increased PTEN expression [72]
HNRNPK	heterogeneous nuclear ribonucleoprotein K	multifunctional RNA-binding protein (RBP), contributes to chromatin remodeling, transcription, splicing, and translation [75]	tumor suppressor or oncogene [76] overexpressed in many cancers: melanoma, PCa, BC (especially in ER+ and/or PR+ BC, including MCF7 cells [75]), lung, CRC [77], HCC, ESCC [78], GC [79]	AT	ER-mediated signaling pathway [75]; high expression was accompanied by high levels of c-Myc in BC [80]	downregulation inhibits lung metastasis [80] and ERα expression [75]
PCBP2	poly(rC)-binding protein 2 isoform b	RNA-binding protein, contributes to transcriptional and translational regulation [81,82]	oncogene, promoter of GC [81], HCC, GBM, BC tissues, and cell lines [83]	AT	overexpression increases cholesterol synthesis and facilitates the stemness of BCSCs via activating PI3K/Akt signaling [82]	depletion decreases GC cells viability and proliferation [81]; inhibits cell proliferation, colony formation, migration, invasion, in vivo tumor growth, and metastasis in BC [83]
MCCC2	methylcrotonyl-CoA carboxylase 2	mitochondrial member of the biotin-dependent carboxylase superfamily	oncogene overexpressed in HCC [84], BC [85], PCa [86], CRC [87]	AT	downregulation of survival-dependent leucine metabolism [84]	knockdown expression reduces cell proliferation, migration, and invasion and glycolysis markers, glucose consumption, lactate secretion, and acetyl-CoA level [84]; promotes apoptosis [86]
UGDH isoform 2	UDP-glucose 6-dehydrogenase	metabolic enzyme associated with mesenchymal-like gene expression [88]	upregulated in epithelial cancers, such as BC [88]; highly metastatic ovarian cancer cell lines [89]; GBM [90]; lung cancer [91]	AT	EMT inhibition, inactivation of ERK/MAPK, metabolic reprogramming, ECM remodeling [88]	knockdown decreases cell motility, invasion, GAGs synthesis, and cell migration [90]; tumor growth, HA production, colony formation [88]; induces cell cycle arrest in G_0_/G_1_ phase [89]
TPI1	triosephosphate isomerase 1	glycolytic enzyme involved in metabolic reprogramming	oncogenic function when translocated to cell nucleus induced by stress condition; overexpressed in multiple cancers: BC tissues and cell lines [92], LUAD [93]	AT	MTORC1_SIGNALING; GLYCOLYSIS; HYPOXIA; EMT [92]	knockdown reduces cell migration, colony formation and xenograft tumor growth [93]
ATP5F1B/ATP5B	adenosine triphosphate synthase F1 subunit beta, mitochondrial precursor	metabolic enzyme in inner mitochondrial membrane, produces ATP via OXPHOS, ectopically expressed on the surface of various cancer cells [94]	participant in carcinogenesis in several tumors, overexpressed in BC, especially in luminal and HER2+ subtypes [94]; plasma membrane of highly invasive cells, including MDA-MB-231 BC cells [95]; GC [96]	AT	OXPHOS; overexpression induces cancer progression via FAK/AKT/MMP2 pathway [96]	overexpression increases intracellular ATP in cancer cells, promoting migration, invasion [95], and proliferation [94]; inhibition suppresses cancer cell metastasis and growth [96]
DLST mitochondrial	dihydrolipoamide S-succinyltransferase	metabolic enzyme	oncogene highly expressed in BC, including MCF7 and MDA-MB-231 BC cell lines [97]; overexpressed in neuroblastoma [98]; TNBC [99]	AT	OXPHOS; ROS [99]	depletion impedes disease initiation and progression; impaires OXPHOS; suppresses growth and TCA-cycle; increases ROS levels; induces apoptosis; decreases burden and invasion [98,99]
FTL	ferritin light chain	iron metabolism regulator [100], hypoxia-responsive gene [101]	oncogene overexpressed in various malignant tumors [101]: GBM cells and serum [100], and CRC tissues and cell lines [102]	AT	interacts with PI3K/Akt, GADD45/JNK, TGF-β signaling, and cell cycle proteins; FTL knockdown decreases the expression of Wnt target genes, cyclin D1, and c-Myc [100]; represses EMT by regulation of Akt/GSK_3_β/β-catenin signaling [101]	knockdown results in inhibition of cancer cell growth and viability by activation of GADD45A expression that impairs cell proliferation (tumor suppressor [103]) [100]; reduces cell migration, invasion, and cancer cell survival rate and increases apoptosis [101]
HYOU1/GRP170/ORP150	hypoxia upregulated 1 (HSP70 protein family)	chaperone with cytoprotective role, involved in protein folding in ER under stressful conditions [104,105]	upregulated in many cancers: BC, PTC, NPC, EOC, KS [105]	AT	silencing promotes OXPHOS and inhibits aerobic glycolysis [105]	silencing suppressed proliferation, migration, and invasion [105]
PRDX6	peroxiredoxin 6	antioxidant enzyme	overexpressed in various cancers [106]; overexpressed in cervical cancer [107]; CRC [108]; MDA-MB-231HM BC cell line [109]	AT	ROS	knockdown inhibits proliferation, migration, and invasion and stimulates apoptosis [107]
NRDC1	nardilysin/N-arginine dibasic convertase 1	nuclear, cytoplasmatic, or cell-surface associated metallo-/zinc-endopeptidase of the M16 family; transcriptional co-regulator [110]; cell-surface receptor for HB-EGF; epigenetic regulatory function [111]	promotion of BC, GC, ESCC, and HCC tissue and serum [110]; overexpressed in CRC [111]	AT	activates IL-6-Signal Transducer and Activator of Transcription 3 (STAT3) signaling [110]; regulates tumor development via HDAC/p53 pathway [111]	involved in cell migration and proliferation [112]; deficiency diminishes tumor size, suppresses carcinogenesis/proliferation [111], spheroid growth, and STAT3 phosphorylation [110]
PRDM5/ PFM2	PRDI-BF1 and RIY domain containing 1	zinc finger protein acts as an epigenetic modifier [113]	tumor suppressor, frequently silenced/downregulated/inactivated by methylation in multiple carcinoma lines: NPC, ESCC [114]; GC [115]; HCC, ovarian, cervical, BC [113,116]	PT	stress-responsive gene, epigenetic regulation of multiple oncogenes, antagonizes Wnt/β-catenin signaling, possible involvement in EMT process [113,114]	knockdown increases cell growth, proliferation, migration, invasion, tumor initiation, and progression [113,114,115]; opposite role in melanoma [116]
DKK1	Dickkopf Wnt signaling pathway inhibitor 1	secretory antagonist of β-catenin-dependent Wnt signaling pathway [117]	tumor suppressor in CC [118] or oncogene abnormally expressed in tumor cells, overexpressed in many cell lines: HCC [119]; lung, BC (incl. in serum), cervical cancers, glioma [117]	AT	WNT_BETA_CATENIN_SIGNALING; overexpression inhibits EMT in CC [118]	knockdown may inhibit migration, invasion, proliferation, cancer stem cell-like proprieties, tumor growth, and angiogenesis; enhances apoptosis and tumor regression [117]
NCKAP1/NAP1	NCK-associated protein 1, isoform CRA_a	member of the Wiskott–Aldrich syndrome protein family member (WASF) regulatory complex (WRC); involved in actin cytoskeleton organization, lamellipodia formation, and cell motility and adhesion [32]	overexpressed in high-grade tumors: BC, PCa, colon cancer [120], NSCLC tissue [32]	AT	WASF3 stability, invasion potential [120]; HSP90-mediated invasion by MMP9 activation, VIM upregulation, and EMT in cancer cells [32]	silencing destabilizes WASF3 complex involved in actin cytoskeletal reorganization, cell movement, and invasion [121]; suppresses invasiveness and metastasis [120]; reduces MMP9 secretion [32]
RPL7A	60S ribosomal protein L7a	ribosomal protein	overexpressed in BC [122], including TNBC, especially metastatic TNBC cells [123], PCa cell lines [124]	AT	activates TRK oncogene [122]; EIF2 signaling [123]	involved in cellular transformation, tumor growth, aggressiveness, and metastasis [122]; blocking RPL7A reduced cell migration and invasion [123]
RPL31	60S ribosomal protein L31	ribosomal protein	overexpressed in PCa [125], CC [126]	AT	knockdown decreases degradation of tumor suppressor p53 and its targets [125]	knockdown enhances levels of p53 and p21, decreases cell growth and cell cycle [125]
RPS3	40S ribosomal protein S3	ribosomal protein involved in ribosomal maturation and translation initiation, DNA damage repair, apoptosis, survival, transcription, and tumorigenesis [127]	oncogenic protein overexpressed in colon adenocarcinoma [127], BC [128]	AT	MYC_TARGETS_v1; ribosome signaling pathway, knockdown increases level of tumor suppressor p53 and induces G1 cell cycle arrest [127]	knockdown promotes ribosomal stress, which impairs ribosomal biogenesis; impedes cell proliferation, invasion, and migration; and increases apoptosis [127]

Abbreviations: ADH—atypical ductal hyperplasia; AJ—APICAL_JUNCTION; AT—anti-tumorigenic; BC—breast cancer; CC—colon cancer; CDH1—E-cadherin; CRC—colorectal cancer; ESCC—esophageal squamous cell carcinoma; DCIS—ductal carcinoma in situ; EMT—epithelial–mesenchymal transition pathway; EOC—epithelial ovarian cancer; ERK—extracellular signal-regulated kinase; FAK—focal adhesion kinase; GADD45—growth arrest and DNA damage-inducible 45; GAGs—glycosaminoglycans; GBM—glioblastoma multiforme; GC—gastric cancer; HA—hyaluronic acid; HB-EGF—heparin-binding EGF-like growth factor; HCC—hepatocellular carcinoma; HM—highly metastatic; IAP—inhibitory of apoptosis proteins; IBC—invasive breast cancer; IMPC—invasive micropapillary carcinoma of the breast; JNK—c-Jun N-terminal kinase; KS—Kaposi sarcoma; LUAD—lung adenocarcinoma; MMP9—metalloproteinase 9; NPC—nasopharyngeal carcinoma; NSCLC—non-small-cell lung cancer; OXPHOS—oxidative phosphorylation pathway; PCa—prostate cancer; PT—pro-tumorigenic; PTC—papillary thyroid carcinoma; PTEN—phosphatase and tensin homolog; ROS—reactive oxygen species pathway; SCLC—small-cell lung cancer; SMAD—family of signal transducers for receptors of the transforming growth factor-beta superfamily; TCA—tricarboxylic acid; TGF-β—transforming growth factor-beta; UPR—unfolded protein response; VIM—vimentin; WAVE—WASP (Wiskott–Aldrich syndrome protein) family Verprolin homolog.

**Table 3:** Pro-tumorigenic (PT) functions of DEPs in JTB^high^ condition in transfected MCF7 BC cell line. X means that the DEP has the corresponding function.

	Biological Processes/ Pathways Involved in Carcinogenesis and Tumor Progression	EMT	Increase in Cell Growth	Cytoskeleton Organization	Inflammatory Response	Cell–cell Adhesion	Chromatin Remodelling	Chromosome/Genomic Instability	Transcription Regulation	Stimulation of Cell Proliferation	Cell Cycle	Increased Cell Invasion	Increased Cell Motility/Migration	Anti-Apoptosis
DEPs														
Pro-tumorigenic (PT) functions														
RBBP4^up^		X					X		X	X	X	X	X	X
SET^up^		X	X				X		X	X		X	X	
ABRACL^up^		X		X						X	X	X	X	
NCKAP1^up^		X	X	X								X	X	
GSN^down^		X		X	X								X	
PRDM5^down^		X	X							X		X	X	

**Table 4:** Anti-tumorigenic (AT) functions of DEPs in JTB^high^ condition in transfected MCF7 BC cell line. X means that the DEP has the corresponding function.

	Biological Processes/ Pathways Involved in Carcinogenesis and Tumor Progression	EMT Inhibition	Proteostasis Regulation	Inhibition of Cell/Tumor Growth	Cytoskeleton Organization	Cell–cell Adhesion	Chromatin Remodelling	Transcription Regulation	Inhibition of Cell Proliferation	Cell Cycle Arrest	Inhibition of Cell Invasion	Inhibition of Cell Motility/Migration	Pro-Apoptosis	Reduced Glycolysis	Increased ROS Production	UPR Increasing	Angiogenesis Inhibition
DEPs																	
Anti-tumorigenic functions (AT)																	
APC^up^						X			X			X					
HINT2^up^		X									X	X	X		X		
UBA1^down^			X						X		X	X	X			X	
YWHAZ^down^				X					X		X		X				
TUBB2A^down^											X	X					
ITGB5^down^		X		X	X	X			X		X	X					X
HSPB1/HSP27^down^													X				
HNRNPK^down^							X	X									
PCBP2^down^				X				X	X		X	X					
MCCC2^down^									X		X	X	X	X			
UGDH^down^		X		X						X	X	X					
TPI1^down^		X		X								X		X			
ATP5F1B^down^				X							X	X	X				
DLST^down^				X							X		X		X		
FTL^down^		X		X					X	X	X	X	X				
HYOU1^down^									X		X	X		X			
PRDX6^down^									X		X	X		X			
NRDC1^down^				X					X		X	X					
DKK1^down^				X					X		X	X	X				X
NCKAP1^down^					X						X	X					
RPL7A^down^				X							X	X					
RPL31^down^				X					X	X	X	X	X				
RPS3^down^				X					X	X	X	X	X				

## Data Availability

Data sharing is not applicable to this article.

## Supplementary Materials

The supporting information can be downloaded at https://www.mdpi.com/article/10.3390/ijms241914714/s1.
