# Supplementary material for: Two-Dimensional Polyacrylamide Gel Electrophoresis Coupled with Nanoliquid Chromatography–Tandem Mass Spectrometry-Based Identification of Differentially Expressed Proteins and Tumorigenic Pathways in the MCF7 Breast Cancer Cell Line Transfected for Jumping Translocation Breakpoint Protein Overexpression
Source: Int J Mol Sci. 2023 Sep 28;24(19):14714. doi: 10.3390/ijms241914714 (PMC10572688; doi:10.3390/ijms241914714)
Supplement: Supplementary file 1 [file ijms-24-14714-s001.zip › ijms-2617073-supplementary.pdf]

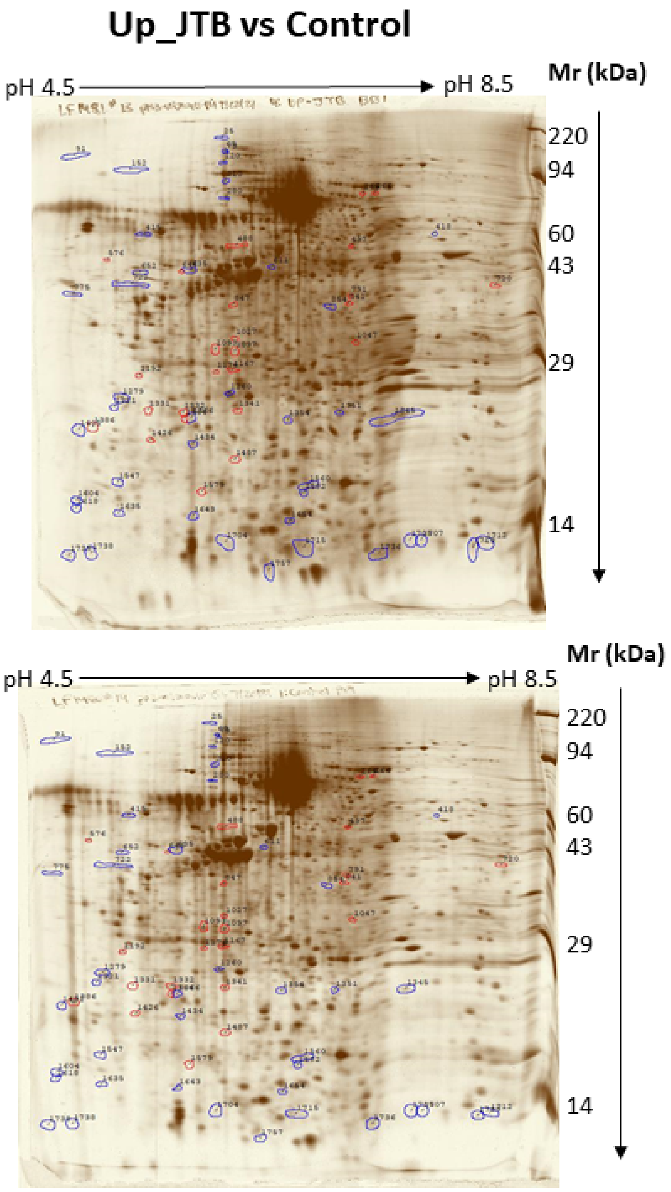

Figure S1. Images of Up_JTB (**top**), Control (**bottom**) silver stained 2D polyacrylamide gels. Polypeptide spots increased in each compared gels (top vs bottom) are shown in blue, while spots decreased are outlined in red.

Up_JTB vs Control

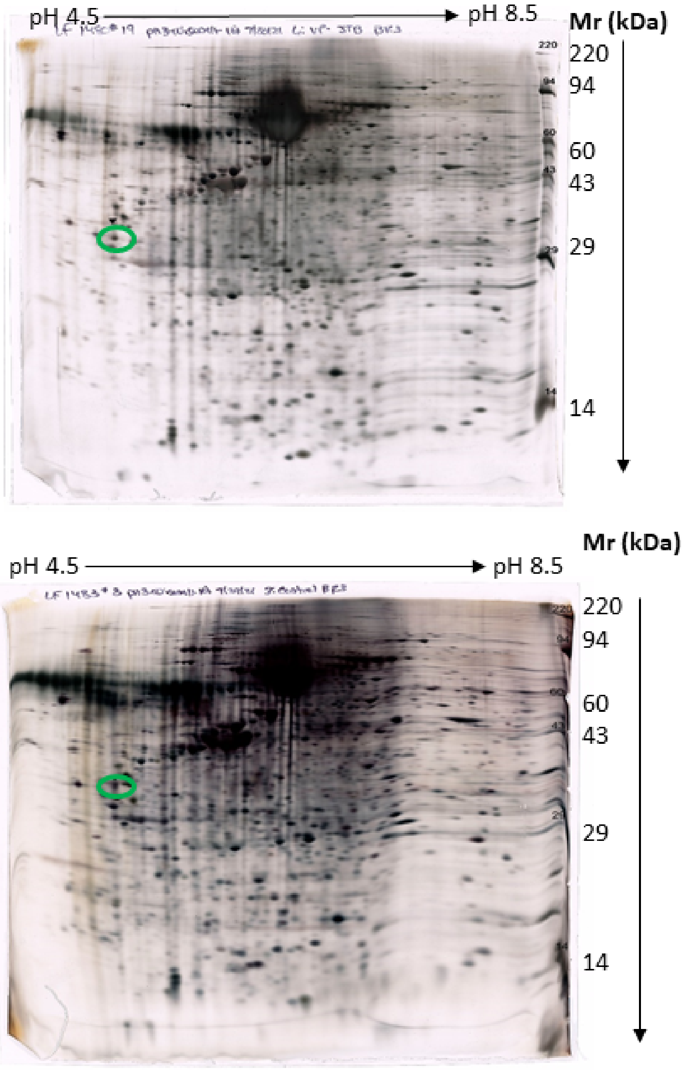

Figure S2. Images of Up_JTB (**top**), Control (**bottom**) silver stained 2D polyacrylamide gels. The circles on each 2D-polyacrylamide gel shows the location of Isoelectric focusing internal standard Tropomyosin of Mw: 33,000 and a pI of 5.2.
